# Bis(quinoline-2-carboxyl­ato-κ^2^ *N*,*O*)lead(II)

**DOI:** 10.1107/S1600536810027509

**Published:** 2010-07-17

**Authors:** Gholamhossein Mohammadnezhad, Ali Reza Ghanbarpour, Mostafa M. Amini, Seik Weng Ng

**Affiliations:** a Department of Chemistry, General Campus, Shahid Beheshti University, Tehran 1983963113, Iran; b Department of Chemistry, University of Malaya, 50603 Kuala Lumpur, Malaysia

## Abstract

The Pb^II^ atom in the title compound, [Pb(C_10_H_6_NO_2_)_2_], is *N*,*O*-chelated by two quinoline-2-carboxyl­ate anions in a distorted Ψ-trigonal–bipyramidal environment; four atoms are connected to the Pb^II^ atom by regular coordination bonds. The structure also features two somewhat long Pb⋯O inter­actions [2.952 (3) and 3.014 (3) Å]. These long inter­actions give rise to a layer coordination polymer having the lead atom in a distorted Ψ-monocapped octa­hedral geometry.

## Related literature

For a related structure, see: Zhang *et al.* (2007 ▶).
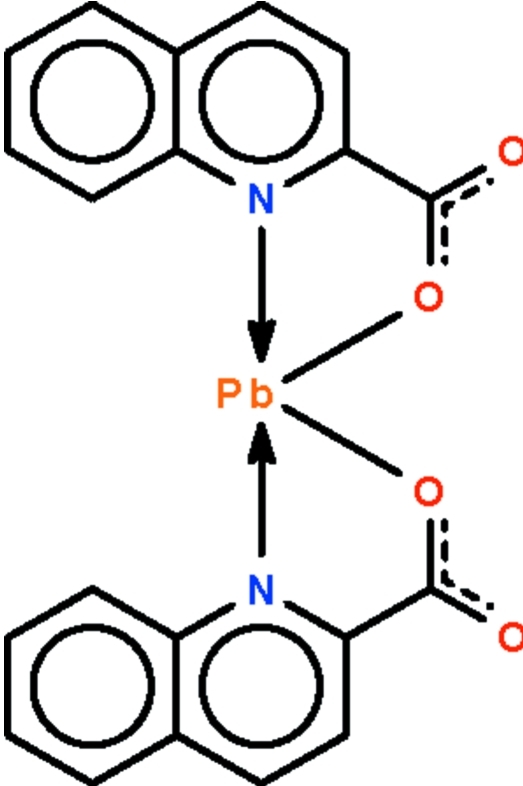


## Experimental

### 

#### Crystal data

[Pb(C_10_H_6_NO_2_)_2_]
*M* *_r_* = 551.51
Monoclinic, 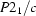

*a* = 16.4510 (15) Å
*b* = 7.2895 (7) Å
*c* = 14.1877 (13) Å
β = 108.981 (1)°
*V* = 1608.9 (3) Å^3^
*Z* = 4
Mo *K*α radiation
μ = 10.52 mm^−1^
*T* = 100 K
0.15 × 0.10 × 0.05 mm

#### Data collection

Bruker SMART APEX diffractometer
Absorption correction: multi-scan (*SADABS*; Sheldrick, 1996 ▶) *T*_min_ = 0.301, *T*_max_ = 0.621
9758 measured reflections
3669 independent reflections
3119 reflections with *I* > 2σ(*I*)
*R*_int_ = 0.033

#### Refinement

*R*[*F* ^2^ > 2σ(*F* ^2^)] = 0.026
*wR*(*F* ^2^) = 0.058
*S* = 1.03
3669 reflections
244 parameters
6 restraints
H-atom parameters constrained
Δρ_max_ = 1.64 e Å^−3^
Δρ_min_ = −0.93 e Å^−3^

### 

Data collection: *APEX2* (Bruker, 2009 ▶); cell refinement: *SAINT* (Bruker, 2009 ▶); data reduction: *SAINT*; program(s) used to solve structure: *SHELXS97* (Sheldrick, 2008 ▶); program(s) used to refine structure: *SHELXL97* (Sheldrick, 2008 ▶); molecular graphics: *X-SEED* (Barbour, 2001 ▶); software used to prepare material for publication: *publCIF* (Westrip, 2010 ▶).

## Supplementary Material

Crystal structure: contains datablocks global, I. DOI: 10.1107/S1600536810027509/xu2797sup1.cif

Structure factors: contains datablocks I. DOI: 10.1107/S1600536810027509/xu2797Isup2.hkl

Additional supplementary materials:  crystallographic information; 3D view; checkCIF report

## Figures and Tables

**Table 1:** Selected bond lengths (Å)

Pb1—O1	2.304 (3)
Pb1—O2^i^	2.952 (3)
Pb1—O3	2.295 (3)
Pb1—O4^ii^	3.014 (3)
Pb1—N1	2.567 (4)
Pb1—N2	2.531 (4)

Symmetry codes: (i) 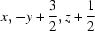; (ii) 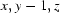.

**Fig. 1.**
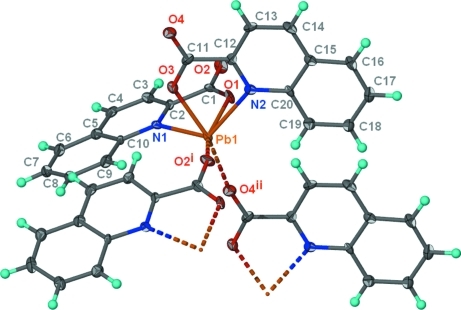
Thermal ellipsoid plot (Barbour, 2001) of the molecule of Pb(C10H6NO2)2 depicted as a part of a layer at the 70% probability level. Hydrogen atoms are drawn as spheres of arbitrary radius. Details of the Pb···O interactions are given in Table 1.

**Fig. 2.**
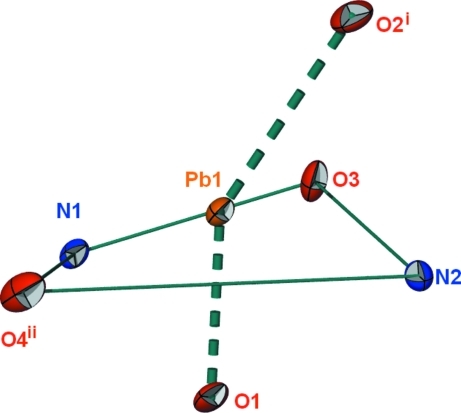
Ψ-Monocapped octahedral geometry of lead. Symmetry codes are given in Table 1.

## supplementary crystallographic information

### Comment

The lone-pair of electrons in lead(II) compounds is usually stereochemically active but the interpretation of the geometry when longer interactions are taken into account is, on the other hand, not usually so rigorous. Lead bis(quinoline-2-carboxylate) crystallizes as a DMSO-coordinated monohydrate but there is no mention of the geometry (Zhang *et al.*, 2007). An examination of the published figure suggests a Ψ-octahedral arrangement.

In the title anhydrous compound (Scheme I, Fig. 1), the lead atom is *N*,*O*-chelated by the carboxylate anions in a Ψ-trigonal bipyramidal environment. However, when two other weaker interactions are considered, the lead atom the exists in a Ψ-monocapped octahedral geometry (Fig. 2).

### Experimental

Lead(II) acetate (1 mmol, 0.38 g), quinoline-2-carboxylic acid (1 mmol, 0.17 g) and sodium nitrite (1 mmol, 0.07 g) were loaded into one arm of a convection tube and both of the arms were filled slowly by methanol. The chemical-bearing arm was immersed in an oil bath kept at 333 K. Crystals were formed on the inside surface of the arm kept at room temperature after a week.

### Refinement

Hydrogen atoms were placed in calculated positions (C–H 0.95 Å) and included in the refinement in the riding model approximation, with *U*(H) set to 1.2*U*_eq_(C). The anisotropic temperature factors of the C10 atom were restrained to be nearly isotropic so as to prevent the atom from going non-positive definite.

The final difference Fourier map had a peak/hole in the vicinity of Pb1.

### Crystal data

**Table:**

[Pb(C_10_H_6_NO_2_)_2_]	*F*(000) = 1040
*M**_r_* = 551.51	*D*_x_ = 2.277 Mg m^−^^3^
Monoclinic, *P*2_1_/*c*	Mo *K*α radiation, λ = 0.71073 Å
Hall symbol: -P 2ybc	Cell parameters from 3819 reflections
*a* = 16.4510 (15) Å	θ = 2.6–28.3°
*b* = 7.2895 (7) Å	µ = 10.52 mm^−^^1^
*c* = 14.1877 (13) Å	*T* = 100 K
β = 108.981 (1)°	Prism, colorless
*V* = 1608.9 (3) Å^3^	0.15 × 0.10 × 0.05 mm
*Z* = 4	

### Data collection

**Table:**

Bruker SMART APEX diffractometer	3669 independent reflections
Radiation source: fine-focus sealed tube	3119 reflections with *I* > 2σ(*I*)
graphite	*R*_int_ = 0.033
ω scans	θ_max_ = 27.5°, θ_min_ = 1.3°
Absorption correction: multi-scan (*SADABS*; Sheldrick, 1996)	*h* = −12→21
*T*_min_ = 0.301, *T*_max_ = 0.621	*k* = −8→9
9758 measured reflections	*l* = −18→18

### Refinement

**Table:**

Refinement on *F*^2^	Primary atom site location: structure-invariant direct methods
Least-squares matrix: full	Secondary atom site location: difference Fourier map
*R*[*F*^2^ > 2σ(*F*^2^)] = 0.026	Hydrogen site location: inferred from neighbouring sites
*wR*(*F*^2^) = 0.058	H-atom parameters constrained
*S* = 1.02	*w* = 1/[σ^2^(*F*_o_^2^) + (0.0278*P*)^2^ + 0.2749*P*] where *P* = (*F*_o_^2^ + 2*F*_c_^2^)/3
3669 reflections	(Δ/σ)_max_ = 0.002
244 parameters	Δρ_max_ = 1.64 e Å^−^^3^
6 restraints	Δρ_min_ = −0.92 e Å^−^^3^

### Fractional atomic coordinates and isotropic or equivalent isotropic displacement parameters (Å^2^)

**Table:**

	*x*	*y*	*z*	*U*_iso_*/*U*_eq_	
Pb1	0.747914 (11)	0.70412 (2)	0.535665 (12)	0.00980 (6)	
O1	0.7953 (2)	0.6837 (4)	0.4000 (2)	0.0147 (7)	
O2	0.7691 (2)	0.6491 (5)	0.2362 (2)	0.0184 (7)	
O3	0.7075 (2)	1.0028 (4)	0.4949 (2)	0.0144 (7)	
O4	0.7419 (2)	1.2990 (4)	0.4913 (3)	0.0196 (7)	
N1	0.6232 (2)	0.6704 (5)	0.3714 (3)	0.0107 (8)	
N2	0.8766 (2)	0.9163 (5)	0.5793 (3)	0.0114 (8)	
C1	0.7447 (3)	0.6590 (6)	0.3111 (3)	0.0126 (10)	
C2	0.6487 (3)	0.6425 (6)	0.2937 (3)	0.0103 (9)	
C3	0.5919 (3)	0.6023 (6)	0.1978 (3)	0.0131 (9)	
H3	0.6131	0.5808	0.1439	0.016*	
C4	0.5058 (3)	0.5948 (6)	0.1835 (3)	0.0138 (9)	
H4	0.4663	0.5701	0.1191	0.017*	
C5	0.4753 (3)	0.6238 (6)	0.2649 (3)	0.0136 (9)	
C6	0.3874 (3)	0.6160 (6)	0.2565 (3)	0.0155 (10)	
H6	0.3456	0.5914	0.1936	0.019*	
C7	0.3621 (3)	0.6437 (6)	0.3383 (4)	0.0176 (10)	
H7	0.3029	0.6358	0.3320	0.021*	
C8	0.4233 (3)	0.6839 (6)	0.4319 (4)	0.0159 (10)	
H8	0.4049	0.7041	0.4880	0.019*	
C9	0.5090 (3)	0.6940 (6)	0.4427 (3)	0.0152 (10)	
H9	0.5496	0.7228	0.5058	0.018*	
C10	0.5372 (3)	0.6615 (6)	0.3599 (3)	0.0087 (9)	
C11	0.7614 (3)	1.1369 (6)	0.5093 (3)	0.0134 (10)	
C12	0.8565 (3)	1.0885 (6)	0.5515 (3)	0.0104 (9)	
C13	0.9195 (3)	1.2240 (6)	0.5577 (3)	0.0138 (10)	
H13	0.9031	1.3456	0.5356	0.017*	
C14	1.0048 (3)	1.1769 (6)	0.5963 (3)	0.0146 (10)	
H14	1.0480	1.2657	0.6002	0.018*	
C15	1.0278 (3)	0.9972 (6)	0.6300 (3)	0.0126 (9)	
C16	1.1143 (3)	0.9387 (6)	0.6713 (3)	0.0155 (10)	
H16	1.1597	1.0243	0.6805	0.019*	
C17	1.1325 (3)	0.7599 (7)	0.6981 (3)	0.0169 (10)	
H17	1.1907	0.7213	0.7233	0.020*	
C18	1.0664 (3)	0.6316 (7)	0.6888 (3)	0.0141 (10)	
H18	1.0803	0.5082	0.7093	0.017*	
C19	0.9823 (3)	0.6841 (6)	0.6505 (3)	0.0136 (10)	
H19	0.9379	0.5973	0.6446	0.016*	
C20	0.9612 (3)	0.8672 (6)	0.6195 (3)	0.0093 (9)	

### Atomic displacement parameters (Å^2^)

**Table:**

	*U*^11^	*U*^22^	*U*^33^	*U*^12^	*U*^13^	*U*^23^
Pb1	0.00792 (9)	0.01030 (9)	0.01183 (8)	−0.00087 (7)	0.00412 (6)	0.00058 (7)
O1	0.0075 (17)	0.0208 (19)	0.0172 (16)	−0.0020 (14)	0.0061 (13)	−0.0022 (13)
O2	0.0192 (19)	0.0226 (18)	0.0175 (16)	0.0033 (15)	0.0117 (14)	−0.0028 (14)
O3	0.0095 (17)	0.0113 (17)	0.0208 (16)	−0.0028 (14)	0.0028 (14)	−0.0009 (13)
O4	0.020 (2)	0.0117 (17)	0.0315 (19)	0.0039 (15)	0.0149 (15)	0.0054 (15)
N1	0.012 (2)	0.0061 (18)	0.0169 (18)	−0.0005 (15)	0.0080 (16)	0.0009 (14)
N2	0.014 (2)	0.0097 (19)	0.0115 (17)	−0.0021 (16)	0.0052 (15)	−0.0014 (14)
C1	0.013 (3)	0.008 (2)	0.020 (2)	0.0001 (18)	0.009 (2)	0.0037 (17)
C2	0.007 (2)	0.006 (2)	0.017 (2)	0.0002 (17)	0.0025 (18)	0.0001 (16)
C3	0.014 (2)	0.013 (2)	0.013 (2)	−0.0032 (19)	0.0045 (18)	−0.0013 (18)
C4	0.015 (3)	0.007 (2)	0.015 (2)	−0.0004 (19)	−0.0009 (18)	0.0007 (17)
C5	0.014 (3)	0.010 (2)	0.016 (2)	0.000 (2)	0.0039 (19)	0.0010 (18)
C6	0.012 (3)	0.013 (2)	0.019 (2)	0.001 (2)	0.0007 (19)	0.0007 (19)
C7	0.010 (3)	0.015 (2)	0.026 (3)	0.000 (2)	0.004 (2)	0.0054 (19)
C8	0.013 (3)	0.012 (2)	0.023 (2)	0.001 (2)	0.007 (2)	0.0040 (19)
C9	0.014 (3)	0.013 (2)	0.016 (2)	0.001 (2)	0.0012 (19)	0.0003 (18)
C10	0.005 (2)	0.005 (2)	0.014 (2)	−0.0006 (16)	0.0015 (17)	0.0007 (15)
C11	0.017 (3)	0.013 (2)	0.012 (2)	0.005 (2)	0.0062 (19)	0.0029 (17)
C12	0.013 (2)	0.012 (2)	0.0087 (19)	0.0034 (18)	0.0066 (17)	−0.0013 (16)
C13	0.019 (3)	0.008 (2)	0.014 (2)	0.001 (2)	0.0065 (19)	0.0034 (17)
C14	0.015 (3)	0.013 (2)	0.017 (2)	−0.0075 (19)	0.0074 (19)	−0.0052 (18)
C15	0.014 (2)	0.013 (2)	0.013 (2)	−0.0028 (19)	0.0073 (18)	−0.0023 (17)
C16	0.010 (2)	0.017 (2)	0.020 (2)	−0.0033 (19)	0.0052 (19)	−0.0039 (19)
C17	0.012 (3)	0.021 (3)	0.016 (2)	0.004 (2)	0.0020 (19)	−0.0007 (18)
C18	0.012 (3)	0.015 (2)	0.015 (2)	0.001 (2)	0.0041 (19)	0.0008 (18)
C19	0.013 (2)	0.014 (2)	0.013 (2)	−0.003 (2)	0.0024 (18)	−0.0011 (18)
C20	0.007 (2)	0.013 (2)	0.0096 (19)	−0.0013 (18)	0.0049 (17)	−0.0030 (17)

### Geometric parameters (Å, °)

**Table:**

Pb1—O1	2.304 (3)	C6—H6	0.9500
Pb1—O2^i^	2.952 (3)	C7—C8	1.412 (7)
Pb1—O3	2.295 (3)	C7—H7	0.9500
Pb1—O4^ii^	3.014 (3)	C8—C9	1.370 (7)
Pb1—N1	2.567 (4)	C8—H8	0.9500
Pb1—N2	2.531 (4)	C9—C10	1.417 (6)
O1—C1	1.279 (6)	C9—H9	0.9500
O2—C1	1.255 (5)	C11—C12	1.525 (6)
O3—C11	1.290 (6)	C12—C13	1.413 (6)
O4—C11	1.229 (5)	C13—C14	1.373 (7)
N1—C2	1.318 (6)	C13—H13	0.9500
N1—C10	1.370 (6)	C14—C15	1.404 (6)
N2—C12	1.325 (5)	C14—H14	0.9500
N2—C20	1.368 (6)	C15—C16	1.417 (6)
C1—C2	1.521 (6)	C15—C20	1.419 (6)
C2—C3	1.408 (6)	C16—C17	1.363 (6)
C3—C4	1.366 (6)	C16—H16	0.9500
C3—H3	0.9500	C17—C18	1.408 (7)
C4—C5	1.416 (6)	C17—H17	0.9500
C4—H4	0.9500	C18—C19	1.366 (6)
C5—C6	1.413 (6)	C18—H18	0.9500
C5—C10	1.427 (6)	C19—C20	1.413 (6)
C6—C7	1.370 (6)	C19—H19	0.9500
			
O3—Pb1—O1	89.98 (11)	C6—C7—H7	119.8
O3—Pb1—N2	68.45 (11)	C8—C7—H7	119.8
O1—Pb1—N2	76.13 (11)	C9—C8—C7	120.6 (5)
O3—Pb1—N1	77.38 (11)	C9—C8—H8	119.7
O1—Pb1—N1	67.90 (11)	C7—C8—H8	119.7
N2—Pb1—N1	129.71 (11)	C8—C9—C10	120.1 (4)
O3—Pb1—O2^i^	80.37 (10)	C8—C9—H9	120.0
O1—Pb1—O2^i^	149.57 (10)	C10—C9—H9	120.0
N2—Pb1—O2^i^	73.48 (10)	N1—C10—C9	119.8 (4)
N1—Pb1—O2^i^	135.90 (11)	N1—C10—C5	120.9 (4)
O3—Pb1—O4^ii^	152.83 (10)	C9—C10—C5	119.3 (4)
O1—Pb1—O4^ii^	76.09 (10)	O4—C11—O3	125.2 (4)
N2—Pb1—O4^ii^	128.16 (11)	O4—C11—C12	118.0 (4)
N1—Pb1—O4^ii^	75.78 (10)	O3—C11—C12	116.8 (4)
O2^i^—Pb1—O4^ii^	122.81 (9)	N2—C12—C13	122.4 (4)
C1—O1—Pb1	122.9 (3)	N2—C12—C11	117.5 (4)
C11—O3—Pb1	123.4 (3)	C13—C12—C11	120.1 (4)
C2—N1—C10	119.3 (4)	C14—C13—C12	118.9 (4)
C2—N1—Pb1	113.3 (3)	C14—C13—H13	120.5
C10—N1—Pb1	127.0 (3)	C12—C13—H13	120.5
C12—N2—C20	119.6 (4)	C13—C14—C15	119.8 (4)
C12—N2—Pb1	113.4 (3)	C13—C14—H14	120.1
C20—N2—Pb1	126.7 (3)	C15—C14—H14	120.1
O2—C1—O1	123.9 (4)	C14—C15—C16	123.0 (4)
O2—C1—C2	117.3 (4)	C14—C15—C20	118.3 (4)
O1—C1—C2	118.8 (4)	C16—C15—C20	118.6 (4)
N1—C2—C3	123.3 (4)	C17—C16—C15	120.2 (4)
N1—C2—C1	116.6 (4)	C17—C16—H16	119.9
C3—C2—C1	120.1 (4)	C15—C16—H16	119.9
C4—C3—C2	118.8 (4)	C16—C17—C18	121.1 (5)
C4—C3—H3	120.6	C16—C17—H17	119.5
C2—C3—H3	120.6	C18—C17—H17	119.5
C3—C4—C5	119.9 (4)	C19—C18—C17	120.1 (5)
C3—C4—H4	120.0	C19—C18—H18	119.9
C5—C4—H4	120.0	C17—C18—H18	119.9
C6—C5—C4	123.3 (4)	C18—C19—C20	120.2 (4)
C6—C5—C10	119.0 (4)	C18—C19—H19	119.9
C4—C5—C10	117.8 (4)	C20—C19—H19	119.9
C7—C6—C5	120.5 (4)	N2—C20—C19	119.4 (4)
C7—C6—H6	119.7	N2—C20—C15	121.0 (4)
C5—C6—H6	119.7	C19—C20—C15	119.6 (4)
C6—C7—C8	120.5 (5)		
			
O3—Pb1—O1—C1	−79.2 (3)	C4—C5—C6—C7	179.4 (5)
N2—Pb1—O1—C1	−147.0 (3)	C10—C5—C6—C7	−0.2 (7)
N1—Pb1—O1—C1	−2.8 (3)	C5—C6—C7—C8	1.2 (7)
O2^i^—Pb1—O1—C1	−149.9 (3)	C6—C7—C8—C9	−0.7 (7)
O4^ii^—Pb1—O1—C1	77.2 (3)	C7—C8—C9—C10	−0.9 (7)
O1—Pb1—O3—C11	−74.7 (3)	C2—N1—C10—C9	−179.1 (4)
N2—Pb1—O3—C11	0.4 (3)	Pb1—N1—C10—C9	7.8 (5)
N1—Pb1—O3—C11	−142.0 (3)	C2—N1—C10—C5	−0.4 (6)
O2^i^—Pb1—O3—C11	76.3 (3)	Pb1—N1—C10—C5	−173.5 (3)
O4^ii^—Pb1—O3—C11	−132.9 (3)	C8—C9—C10—N1	−179.5 (4)
O3—Pb1—N1—C2	100.8 (3)	C8—C9—C10—C5	1.9 (6)
O1—Pb1—N1—C2	5.6 (3)	C6—C5—C10—N1	−180.0 (4)
N2—Pb1—N1—C2	53.2 (3)	C4—C5—C10—N1	0.4 (6)
O2^i^—Pb1—N1—C2	162.2 (3)	C6—C5—C10—C9	−1.3 (6)
O4^ii^—Pb1—N1—C2	−75.0 (3)	C4—C5—C10—C9	179.1 (4)
O3—Pb1—N1—C10	−85.8 (3)	Pb1—O3—C11—O4	−177.6 (3)
O1—Pb1—N1—C10	179.0 (4)	Pb1—O3—C11—C12	2.8 (5)
N2—Pb1—N1—C10	−133.3 (3)	C20—N2—C12—C13	2.8 (6)
O2^i^—Pb1—N1—C10	−24.3 (4)	Pb1—N2—C12—C13	−172.1 (3)
O4^ii^—Pb1—N1—C10	98.5 (3)	C20—N2—C12—C11	−178.7 (3)
O3—Pb1—N2—C12	−3.8 (3)	Pb1—N2—C12—C11	6.5 (4)
O1—Pb1—N2—C12	91.8 (3)	O4—C11—C12—N2	173.9 (4)
N1—Pb1—N2—C12	46.9 (3)	O3—C11—C12—N2	−6.5 (6)
O2^i^—Pb1—N2—C12	−89.8 (3)	O4—C11—C12—C13	−7.6 (6)
O4^ii^—Pb1—N2—C12	151.2 (2)	O3—C11—C12—C13	172.1 (4)
O3—Pb1—N2—C20	−178.2 (3)	N2—C12—C13—C14	−1.7 (6)
O1—Pb1—N2—C20	−82.6 (3)	C11—C12—C13—C14	179.8 (4)
N1—Pb1—N2—C20	−127.5 (3)	C12—C13—C14—C15	−0.9 (7)
O2^i^—Pb1—N2—C20	95.9 (3)	C13—C14—C15—C16	−179.5 (4)
O4^ii^—Pb1—N2—C20	−23.2 (4)	C13—C14—C15—C20	2.3 (6)
Pb1—O1—C1—O2	179.4 (3)	C14—C15—C16—C17	−176.7 (4)
Pb1—O1—C1—C2	0.1 (5)	C20—C15—C16—C17	1.5 (6)
C10—N1—C2—C3	−0.4 (6)	C15—C16—C17—C18	−2.4 (7)
Pb1—N1—C2—C3	173.6 (3)	C16—C17—C18—C19	1.5 (7)
C10—N1—C2—C1	178.5 (4)	C17—C18—C19—C20	0.3 (7)
Pb1—N1—C2—C1	−7.5 (5)	C12—N2—C20—C19	179.0 (4)
O2—C1—C2—N1	−173.8 (4)	Pb1—N2—C20—C19	−6.9 (5)
O1—C1—C2—N1	5.5 (6)	C12—N2—C20—C15	−1.3 (6)
O2—C1—C2—C3	5.2 (6)	Pb1—N2—C20—C15	172.8 (3)
O1—C1—C2—C3	−175.5 (4)	C18—C19—C20—N2	178.6 (4)
N1—C2—C3—C4	1.2 (7)	C18—C19—C20—C15	−1.1 (6)
C1—C2—C3—C4	−177.7 (4)	C14—C15—C20—N2	−1.3 (6)
C2—C3—C4—C5	−1.1 (7)	C16—C15—C20—N2	−179.5 (4)
C3—C4—C5—C6	−179.2 (4)	C14—C15—C20—C19	178.5 (4)
C3—C4—C5—C10	0.4 (7)	C16—C15—C20—C19	0.2 (6)

Symmetry codes: (i) *x*, −*y*+3/2, *z*+1/2; (ii) *x*, *y*−1, *z*.
